# *Aspergillus niger* Environmental Isolates and Their Specific Diversity Through Metabolite Profiling

**DOI:** 10.3389/fmicb.2021.658010

**Published:** 2021-06-23

**Authors:** Alexandra Šimonovičová, Hana Vojtková, Sanja Nosalj, Elena Piecková, Hana Švehláková, Lucia Kraková, Hana Drahovská, Barbara Stalmachová, Kateřina Kučová, Domenico Pangallo

**Affiliations:** ^1^Department of Soil Science, Faculty of Natural Sciences, Comenius University, Bratislava, Slovakia; ^2^Department of Environmental Engineering, Faculty of Mining and Geology, VSB – Technical University of Ostrava, Ostrava, Czechia; ^3^Department of Microbiology, Slovak Medical University in Bratislava, Bratislava, Slovakia; ^4^Institute of Molecular Biology, Slovak Academy of Sciences, Bratislava, Slovakia; ^5^Department of Molecular Biology, Faculty of Natural Sciences, Comenius University, Bratislava, Slovakia

**Keywords:** *Aspergillus niger* environmental isolates, molecular analyses, Biolog FF Microplate^TM^, multi-criteria data analysis, extrolite profile

## Abstract

We present a biological profile of 16 *Aspergillus niger* environmental isolates from different types of soils and solid substrates across a pH range, from an ultra-acidic (<3.5) to a very strongly alkaline (>9.0) environment. The soils and solid substrates also differ in varying degrees of anthropic pollution, which in most cases is caused by several centuries of mining activity at old mining sites, sludge beds, ore deposits, stream sediments, and coal dust. The values of toxic elements (As, Sb, Zn, Cu, Pb) very often exceed the limit values. The isolates possess different macro- and micromorphological features. All the identifications of *Aspergillus niger* isolates were confirmed by molecular PCR analysis and their similarity was expressed by RAMP analysis. The biochemical profile of isolates based on FF-MicroPlate tests from the Biolog system showed identical biochemical reactions in 50 tests, while in 46 tests the utilisation reactions differed. The highest similarity of strains isolated from substrates with the same pH, as well as the most suitable biochemical tests for analysis of the phenotypic similarity of isolated strains, were confirmed when evaluating the biochemical profile using multicriterial analysis in the Canoco program. The isolates were screened for mycotoxin production by thin-layer chromatography (TLC), as well. Two of them were able to synthesise ochratoxin A, while none produced fumonisins under experimental conditions. Presence of toxic compounds in contaminated sites may affect environmental microscopic fungi and cause the genome alteration, which may result in changes of their physiology, including the production of different (secondary) metabolites, such as mycotoxins.

## Introduction

*Aspergillus niger* is a cosmopolitan representative of microscopic filamentous fungi. Although the main source of this strain is soil, it frequently occurs in various other sources, such as historical and archaeological objects (Abdel-Kareem, 2010; Pangallo et al., 2012; Aldosari et al., 2019; Geweely et al., 2019) or indoor environments (Egbuta et al., 2017; Omar et al., 2018). The ability of *Aspergillus niger* to produce substances of various types, such as low molecular weight organic acids (e.g., gluconic, citric, itaconic, oxalic, malic, acetic, lactic, and others), enzymes (e.g., amylase, aryl-phosphatase, β-glucosidase, cellulase, lipase, and others) as well as other products of metabolism, has great use not only in the food, medicine, pharmaceutical, and chemical industries but also in mineral biotechnology (Steiger et al., 2013; Akhtar et al., 2014; Utobo and Tewari, 2014; Adeleke et al., 2017; Odoni et al., 2017). According to Esteban et al. (2006), up to 6% of *A. niger* strains synthesise the mycotoxin ochratoxin A, and its degree of production depends on environmental factors, such as the substrate or cultivation medium type, cultivation time, etc. Foodborne *A. niger* strains are also able to produce the mycotoxins fumonisins, namely fumonisin B1 and B2 (Noonim et al., 2009; Mikušová et al., 2020). The cultivation medium notably affects fumonisin synthesis, as is the case with ochratoxin A production (Frisvad et al., 2007). The ability to synthesise mycotoxins always depends on the expression of the genome of a particular strain of toxic fungal species (Susca et al., 2016).

These metabolic products are mainly used in the biodegradation processes of environmental pollutants, such as crude oil and its products found in industrial water and wastewater (Gulzar et al., 2017). The biosorption and bioleaching of heavy metals and toxic elements from solutions and soils are key processes in bioremediation (Acosta-Rodriguez et al., 2018; Xinhui et al., 2018). This means the treatment of contaminated water, soil and subsurface material by altering environmental conditions to stimulate the growth of microorganisms can degrade the target pollutants. Among the most commonly applied species or strains or certain types of microscopic filamentous fungi in these processes were those that were isolated from the contaminated environment. They are expected to be more effective than species or strains isolated from an uncontaminated environmental source (Hindersah et al., 2018). According to Chávez et al. (2015), the potential for the discovery of novel metabolites in microscopic filamentous fungi is huge. In this context, fungi thriving in harsh environments are of particular interest, since they are outstanding producers of unusual chemical structures.

The Biolog technique, or Biolog FF MicroPlates, has been used in microbiology for more than fifteen years. Klingler et al. (1992) were the first to do so. The Biolog technique was also used in ecological studies to estimate the metabolic potential of microbial communities from rhizospheric soil (Stefanowicz, 2006), substrate utilisation, growth, secondary metabolite, and the antimicrobial profiles of some fungal cultures important for the microbial drug discovery program (Singh, 2009). The Biolog system was used to enable the evaluation of the metabolic profile diversity of microbial populations in environmental samples, which reflects the state of their activity (Gryta et al., 2014; Šimonovičová et al., 2020). Twelve *Aspergillus* sp. strains were also characterised using Biolog FF MicroPlates to obtain data on C-substrate utilisation and mitochondrial activity (Rola et al., 2015) in testing the functional diversity of root-colonising endophytic fungi (Knapp and Kovács, 2016). Biolog FF Microplate system also identified soil fungal isolates from forest soil for the recovery of flooded soil (Aziz and Zainol, 2018).

The aim of our study was to show the biological profile of 16 *Aspergillus niger* environmental isolates focusing on the biochemical profiling and physiology of the strains isolated from different types of soils and solid substrates, such as stream sediments, ash layer, coal dust and the surface of artificial adamite [Zn_2_(AsO_4_)(OH)], by using the Biolog FF MicroPlate system. Besides metal contamination, the substrates also differed from each other in pH across a range from an ultra-acidic (<3.5) to a very strongly alkaline environment (>9.0).

Contamination of some localities is more than 700 years old and dates back to mining times, so we expected also a changes in the genome associated with the microstructure of study strains, including the production of different (secondary) metabolites, such as mycotoxins.

The biochemical profile of strains based on FF-microplate tests from the Biolog system showed identical biochemical reactions in 50 tests, while in 46 tests the utilisation reactions differed. Data were processed using the PCA method (Principal Component Analysis) in the CANOCO 5 program (Braak and Smilauer, 2012).

In terms of physiological and biochemical properties is *Aspergillus niger* known as a very good producer of mycotoxins. Therefore all our investigated strains were screened for their ability to produce ochratoxin A (OTA) and fumonisin B1 (FB1) in vitro by rapid thin-layer chromatography (TLC) (Filtenborg et al., 1989; Nelson et al., 1993; Nielsen et al., 2020).

The species *Aspergillus niger*, along with 16 environmental strains that we isolated, was shown to be a suitable model organism in comparing variable experimental methods with the aim of identifying their common, respectively, different characteristics depending on the environment of origin.

## Materials and Methods

### *Aspergillus niger* Environmental Isolates

**FIGURE 1:**
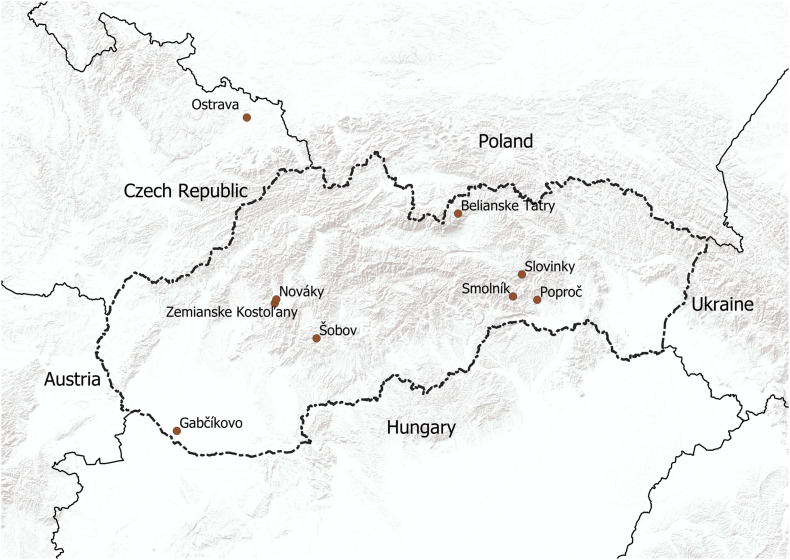
Map showing the sampling localities in Slovakia and the Czechia.

Sixteen *Aspergillus niger* (*A*. *niger*) strains were isolated from different types of soils (samples 1, 3, 4, 7, 8, 10, 12–l4), coal dust (sample 2), stream sediments (samples 5 and 6), an ash layer (sample 11), a tailing pond (samples 9 and 15) and a surface of artificial adamite (sample 16) (Figure 1). The samples from all solid substrates were taken from three places at each site. Then in laboratory by mixing an average sample was prepared and analysed. From the surface of artificial adamite was *A*. *niger* isolated by using a SWABS swab (1660, Dispolab Brno, Czechia) and subsequent cultivated on Sabouraud Dextrose Agar (M063), HiMedia Labs., Mumbai, India). The chemical characteristics of the localities and substrates from which *A*. *niger* strains were isolated are shown in Table 1.

**TABLE 1:** *Aspergillus niger* environmental isolates obtained from different type of soils and solid sources and its chemical characteristics.

Order of samples according to pH range	The range of pH according to Čurlík et al., 2003	An fungal strains	Substrates of studied localities and its chemical characteristics	ITS-product sequence similarity	References
1.		An–S	Banská Štiavnica—Šobov; Dystric Cambisol (contaminated and eroded) without vegetation; pH 3.12; %C_ox_ 0.49; *Al 727–506 mg/kg	AM270051 99.6%	Šimonovičová et al., 2013, 2019
2.	<3.5 Ultra acidic	An–N	Nováky; coal dust; pH 3.32; %C_ox_ 39; *As 400 mg/kg; Mn 302.4 mg/kg; *Zn 21.4 mg/kg	AM270051 99.8%	Šimonovičová et al., 2013, 2019

3.	3.5–4.4 Extreme acidic	An–Pop 4	Poproč; Technosol; pH 3.85; % C_ox_ 0.1; *As 25 mg/kg; *Sb 5,825 mg/kg; *Zn 150 mg/kg; *Cu 60 mg/kg; Pb 70 mg/kg; Hg 0.5 mg/kg	KX901281 100%	Šimonovičová et al., 2019

4.	4.5–5.0 Very strong acidic	An–Pop 1	Poproč; Haplic Leptosol; pH 4.52; % C_ox_ 2.62; *As 25 mg/kg; *Sb 1,022 mg/kg; *Zn 150 mg/kg; Cu 60 mg/kg; Pb 70 mg/kg; Hg 0.5 mg/kg	KX253667 100%	Šimonovičová et al., 2019

5.		An–P	Pezinok; stream sediment of Blatina river; pH 5.25; %C_ox_ 7.2; *As 363 mg/kg; Sb 93 mg/kg; Fe 82.8 mg/kg; Al 5.5 %	AM270051 99.8%	Šimonovičová et al., 2013
6.	5.1–5.5 Strong acidic	An–Sm	Smolník; stream sediment; pH 5.4; % C_ox_ 0.7; *Mg 344 mg/l; *Fe 463 mg/l; *Mn 36.5 mg/l; *Al 107 mg/l; *Cu 3,263 μg/l; *Zn 12,600 μg/l; *Cd 15 μg/l	AM270051 100%	Šimonovičová et al., 2019
7.		An–Kmi	Cambisol (Calcaric) at a foot of a Kýčera hill in Belianske Tatry; pH 5.4; % C_ox_ 0.4	KY657577 100%	Bedrna et al., 2010

8.	5.6–6.0 Medium acidic	An–Pop 5	Poproč; Technosol; pH 6.05; %C_ox_ 0.14; *As 200 mg/kg; *Sb 2,099 mg/kg; *Zn 200 mg/kg; Cu 70 mg/kg; *Pb 115 mg/kg; Hg 0.75 mg/kg	KY657577 100%	Šimonovičová et al., 2019

No sample	6.1–6.5 Week acid	No sample			

9.	6.6–7.3 Neutral	An–L18	Lagoon Ostrava, Czechia; sludge from oil refining and other chemical processing; pH 6.85; NEL 201 000 mg/kg; PAH C_10_-C_40_ 121 000 mg/kg; *Cr 182 mg/kg; *Cu 2 102 mg/kg; *Zn 6 946 mg/kg; *Ba 3 652 mg/kg; *Pb 4 066 mg/kg	KU702453 100%	This publication

10.		An–Pop 3	Poproč; Haplic Leptosol; pH 7.45; %C_ox_ 0.91; *As 25 mg/kg; *Sb 12,200 mg/kg; *Zn 150 mg/kg; Cu 60 mg/kg; Pb 70 mg/kg; Hg 0,5 mg/kg	KX426976 100%	Šimonovičová et al., 2019
11.	7.4–7.8 Slightly alkali	An–ZK	Zemianske Kostol’any; ash layer; pH 7.51; %C_ox_ 1.38; *As 634 μg/g; Zn 47 μg/g; Hg 0.47 μg/g	KF031033 100%	Šimonovičová et al., 2019
12.		An–G	Eutric Fluvisol from a floodplain forest in Gabčíkovo; pH 7.7; %C_ox_ 2.7	AM270051 100%	Šimonovičová et al., 2013, 2019

13.	7.9–8.4 Medium alkali	An–KD	Kuwajt; soil sample from desert; pH 8.25	KX253667 100%	This publication
14.		An–KF	Kuwajt; soil sample from a farm; pH 8.49	KX253667 100%	This publication
15.	8.5–9.0 Strong alkali	An–SL	Slovinky; Technosol; pH 8.6; %C_ox_ 0.8; *As 511 mg/kg; *Cu 8,186 mg/kg; *Zn 25,108 mg/kg; *Pb 2,964 mg/kg; *Mn 2,647 mg/kg; *Cd 8.76 mg/kg	KF031033 100%	Šimonovičová et al., 2019

no sample	>9.0 Very strong alkali	No sample			

16.		An–A	Surface of artificial adamite [Zn_2_(AsO_4_)(OH)]	MT597437 100%	Kolenčík et al., 2017

*Exceeded values of elements; NEL, non-polar extractables; PAH, polyaromatic hydrocarbons, hydrocarbons C_10_ – C_40_ is an indicator for the determination of oil pollution, it is the amount of extractable non-polar hydrocarbons of petroleum and non-petroleum origin with 10–40 carbons per molecule contained in the matrix.

The metallic element composition of the samples from the Banská Štiavnica—Šobov, Pezinok, Nováky, and Smolník localities were analysed by the EL spol. Ltd. accredited test labs in Spišská Nová Ves (Slovakia). Samples from the Poproč, Zemianske Kostol’any, and Slovinky localities were analysed for their metal content at ACME Analytical Laboratories Ltd. (Vancouver, Canada) by ICP-ES or ICP-MS (Šimonovičová et al., 2019). Persistent toxic substances in the Ostrava Lagoons locality were analysed in sludge samples (mg/kg of dry weight) using several methods. XRF technology—X-ray fluorescence spectrometry with high sensitivity on the S8 TIGER instrument (Bruker Co., United States) was used for the analysis of metal and metalloid residues. Organic pollution analyses were performed at ALS Czech Republic, Ltd. (a testing laboratory accredited according to ČSN (Czech National Standard) EN ISO/IEC 17025:2005).

All strains were isolated from mixed cultures of fungi by the dilution plate method on SDA (Sabouraud Dextrose Agar, FyHimedia Laboratories, Mumbai, India) at a laboratory temperature of 25°C for 5–7 days (Šimonovičová et al., 2013, 2017, 2019). All isolates were assigned to *A*. *niger* according to molecular analyses and according to the name of the locality, as shown in Table 1. All strains are deposited on SDA in the collection of filamentous fungi in the Department of Soil Science at the Faculty of Natural Sciences, Comenius University in Bratislava (Slovakia). The ITS sequences of the *Aspergillus niger* isolates were deposited in GenBank under accession numbers MW739953-MW739968.

### Macro and Micromorphological Features of the *A*. *niger* Environmental Isolates

**FIGURE 2:**
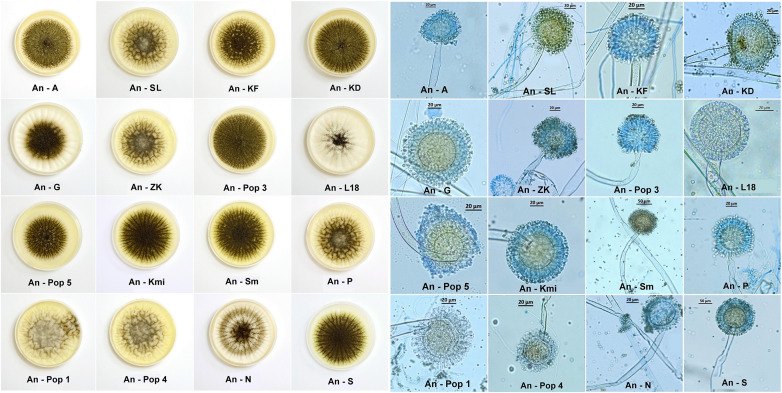
Macromorphological features of *Aspergillus niger* environmental isolates on the fifth day of cultivation on SDA agar in Petri dishes with a diameter of 6 cm. Colonies form different diameters and also sporulation depending on environmental contamination (left side). Micromorphological features of *Aspergillus niger* environmental isolates on the fifth day in a drop of lactic acid enriched with cotton blue stain (0.01%). The figures show deformations of conidiophores, such as swelling, narrowing or deformation of the conidial head (vesiculum). We assumed that these deformations can be caused by long term contaminated environment (right side).

The macromorphological features, i.e., the diameter of colonies and degree of sporulation, were observed visually on the fifth day of cultivation on SDA in Petri dishes and had a diameter of 6 cm in three repetitions. The micromorphology figures were made with a Canon IXUS 16.1 megapixels camera (Japan). Also, micromorphological features (Figure 2) were observed under an Axio Scope A1 Carl Zeiss Jena light microscope on the fifth day of cultivation in a drop of lactic acid enriched with a cotton blue stain (0.01%).

### Genotypic Profile of *Aspergillus niger* Environmental Isolates

The DNA of *A. niger* isolates was extracted using a DNeasy Plant mini purification kit (Qiagen, Hilden, Germany) according to the manufacturer’s instructions. The Random Amplified Microsatellite Polymorphism-Polymerase Chain Reaction (RAMP-PCR) was performed according to the protocol of Pangallo et al. (2012). The PCR reaction mixture (25 μL) contained: 2 U (0.4 μL) of SuperHotTag DNA polymerase (Bioron), 1× reaction buffer (2.5 μL), 2.4 mmol^–*l*^ MgCl_2_ (0.6 μL), 200 μmol^–*l*^ dNTP (0.4 μL), 60 pmol (0.4 μL) of each primer T14 (AAT GCC GCA G), and K7 (CAA CTC TCT CTC TCT), 17.3 μL of deionised water and approximately 60 ng (3 μL) of DNA. The PCR amplification program included: a denaturation step at 95°C for 5 min; 30 cycles: 95°C 45 s; 30°C for 60 s with ramping 0.1°C/s to 50°C, 50°C for 60 s with ramping 0.1°C/s to 68°C, 68°C for 180 s, and a final polymerisation at 68°C for 10 min. Five microlitres of RAMP-PCR amplicon were separated on 1.8% agarose gel for 4.5 h at 2.3 V cm^–1^ in TAE buffer (Trid-acetate-EDTA). The gel was stained with ethidium bromide, visualised under UV light and digitalised.

The RAMP profiles were analysed according to BioNumerics software ver. 6 (Applied Maths, Kortrijk, Belgium) using Pearson correlation and UPGMA clustering.

### Physiological Profile of *Aspergillus niger* Environmental Isolates

The biochemical profile of *Aspergillus niger* environmental isolates was obtained using the Biolog MicroStation test system (MicroLog3 ver. 4.20.05, Biolog Inc., United States). By using a 96-well Biolog FF MicroPlate, unique phenotypic fingerprinting can be obtained for individual strains; this is a manifestation of their catabolic potential. Metabolic reactions, dependent on the production of suitable enzymes for the oxidation of the tested substrates, are quantified by the formation of tetrazolium dye (Bochner, 2009). These results, as fingerprint reaction patterns, provide a lot of information about the individual differences in the metabolic properties of each fungus tested.

All *A. niger* environmental isolates were cultured in tubes on 2% slants of MEA (Malt Extract Agar HiMedia Laboratories, Mumbai, India) at 26°C for 5–7 days. Conidia were then inoculated into FF-IF inoculating fluid (72106 FF-IF inoculating fluid, Biolog, United States) according to the Biolog protocol and the density was adjusted to the recommended value of 65%, which was measured using a turbidimeter. Subsequently, we inoculated 100 μL of conidia suspension onto plates containing a pre-set of test substrates. We incubated the microplates for 72–168 h and then evaluated them using the Biolog MicroStation system.

### Mycotoxins of *Aspergillus niger* Environmental Isolates

Strains of *A. niger* were cultivated on MEA (Malt Extract Agar, HiMedia Laboratories, Mumbai, India) agar plates (thickness of the solid medium 0.5 cm) at room temperature for 14 days.

Rings of the fungal cultures (*d* = 1 cm) were placed onto the start line on the TLC silica gel plate (Merck TLC aluminium silica gel, 20 × 20 cm, Merck KGaA, Darmstadt, Germany). The medium side of the rings placed onto the silica gel enables diffusion of the fungal exotoxins (excreted by the fungus into its growing medium, here MEA) into the sorbent (here silica gel), while the biomass side soaked with two drops of extraction mixture methanol/water (80:20, disrupting the fungal cell wall and extracting the toxicants present inside the cell) was used to detect fungal endotoxins (kept in fungal cells). Griseofulvin (10 μL of a solution 100 μg/ml chloroform) (Sigma Aldrich, St. Louis, United States) was applied as the standard. Dried TLC plates were eluted in a TEF mixture (toluene/ethyl acetate/formic acid 90%, 5:4:1) (Merck KGaA, Darmstadt, Germany) at the exotoxin side and in a CAP solution (chloroform/acetone/isopropanol, 85:15:20) at the side of endotoxins in preparation cuvettes (Camag, Muttenz, Switzerland) (Nielsen et al., 2020).

Upon completion of both elutions, the spots of extrolites (metabolites), including potentially present mycotoxins, were visualised. The production of OTA was confirmed, as presented by Filtenborg et al. (1989). The dried TLC plate was exposed to NH_3_ (Lachema, Brno, Czechia) vapour, and the spots were observed under UV light (254 nm) in the TLC cabinet Camag TLC Visualiser 2 (Camag, Muttenz, Switzerland). OTA spots gave blue-green and the griseofulvin spot blue fluorescence.

FB1 was detected using the method developed by Nelson et al. (1993). TLC plates were eluted in a mixture of chloroform/methanol/acetic acid (6:3:1). Dried plates were sprayed with 0.5% p-anisaldehyde in a mixture of ethanol/acetic acid/sulphuric acid (17:2:1) and heated at 140°C for 2–3 min. FB1 appears as lilac-deep lilac spots, the standard as a blue spot.

**FIGURE 3:**
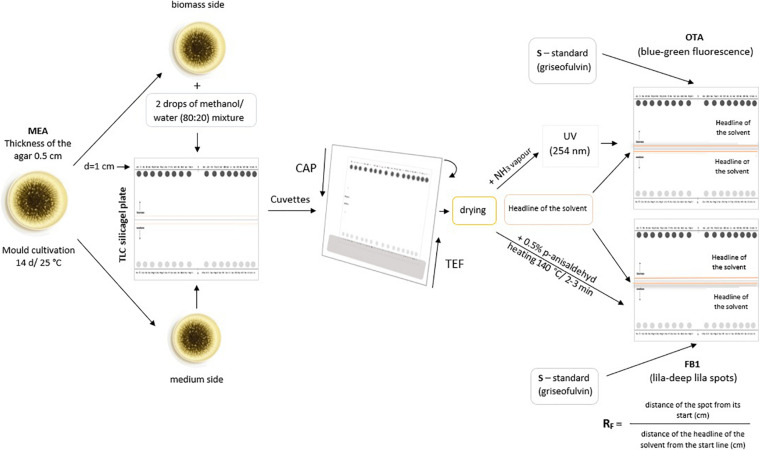
Diagram of the TLC analysis of mycotoxins synthesised by the tested *Aspergillus niger* strains. MEA, malt extract agar; CAP, chloroform/acetone/isopropanol, 85:15:20; TEF, toluene/ethyl acetate/formic acid 90%, 5:4:1; OTA, ochratoxin A; FB1, fumonisin B1; Rf, retention factor.

All the spots observed were characterised by their retention factors (RF). The ratio of RF of the spot analysed to the RF of the standard griseofulvin (always considered equal to one) was proving the particular mycotoxin production by comparison to the database of these ratio factors according to Filtenborg et al. (1989). The complex method is graphically documented in Figure 3.

### Data Processing

Multicriteria analysis (Principal Component Analysis—PCA) in the Canoco 5 program (Braak and Smilauer, 2012) was used to analyse the phenotypic similarity of the strains based on biochemical parameters obtained by the Biolog system, which confirmed the highest similarities of strains isolated from substrates with the same pH and the most suitable biochemical tests for determining the similarity of the tested strains. The similarity among the strains was determined using the Pearson correlation coefficient. Cluster analysis was performed using UPGMA clustering.

## Results and Discussion

### The Characteristics of Localities

All the *Aspergillus niger* isolates from environmental samples from Slovakia, Czechia (Figure 1) and Kuwait used in our study point out to the extreme adaptability of this species across a wide pH range and its high tolerance to metal contamination, despite changes in the macro- and microstructure (Figure 2). Among different type of soils, *Aspergillus niger* strains were isolated from Dystric Cambisol (contaminated and eroded), Technosol, Haplic Leptosol, Cambisol (Calcaric), and Eutric Fluvisol, all of which, the last two excepted, were contaminated with heavy metals and toxic elements exceeding the limit values for soils according to Act No. 220 (2004) in Slovakia. This contamination is in most cases caused by several centuries of mining activities, such as quartzite quarrying since 1956 at the Banská Štiavnica—Šobov locality, exploitation of Sb and Au from the seventeenth century at the Poproč locality, or from iron ore mining since the fifteenth century at the Slovinky locality. It can be assumed that both soil samples from Kuwait are also contaminated with petroleum substances due to the discovery of petroleum in 1930 and the rapid development of the oil industry. According to Cho et al. (1997) in the Kuwajt desert remains a huge amount of soil contaminated by oil. It was also confirmed by Asem et al. (2016) which studied the impact of crude oil pollution on different types of soils in Kuwajt and the contamination was detected up to a soil depth of more than 2 m. Also, all solid substrates are among the contaminated. These include coal dust from the coal mine at the Nováky locality, stream sediments influenced by Sb mining activities from the nineteenth century at the Pezinok locality, the ash slurry present at the locality Zemianske Kostol’any due to a dam failure in 1965, and stream sediment at the Smolník locality, influenced by acid-mining drainage from one of the richest Cu-Fe ore mines in Slovakia, which had its highest activity in fourteenth century. The Ostrava Lagoons locality in the Czechia was created in 1965 and contains sludge and dumped waste from oil refining and separation processes. We did not expect contamination at the Belianske Tatry and Gabčíkovo localities. The Belianske Tatry mountains are part of the Tatra National Park and Gabčíkovo is a cultivated willow-poplar stand on the site of a floodplain forest. The An-G isolate was used as a comparative for further analyses (Table 1).

### Genotype Profile of *Aspergillus niger* Environmental Isolates

**FIGURE 4:**
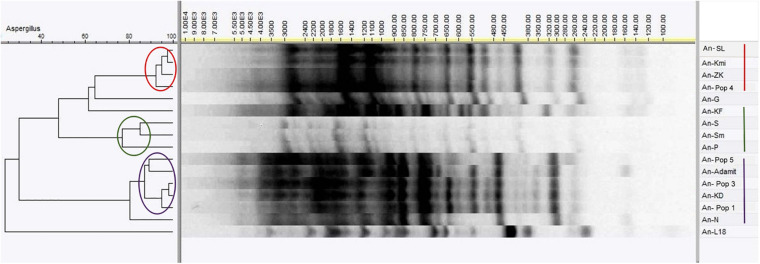
RAMP analysis of *Aspergillus niger* environmental isolates using Pearson correlation and UPGMA clustering.

All environmental isolates of *Aspergillus niger* were compared on the DNA level by the RAMP method. A cluster analysis of RAMP profiles separated the strains into three main groups and several unique branches (Figure 4). The first group contained the An-SL, An-Kmi, An-ZK, and An-Pop 4 strains possessing a similarity of more than 90%. Six strains (An-Pop 5, An-A, An-Pop 3, An-KD, An-Pop 1, and An-N) were included in the largest cluster, with a similarity of 80–95%. The third cluster consisted of the An-S, An-Sm and An-P strains, with a similarity of 75–85%. Two strains (An-G and An-KF) had unique RAMP profiles, but both were clustered among other *A*. *niger* isolates. The An-L18 strain from the Ostrava Lagoon was the most different and clustered outside the other strains. The strain clustering did not correspond with the pH values or the contamination of the environments. In the subsequent experiments we used biochemical profiling for a more considerable comparison of the analysed strains.

### Physiological Profile of *Aspergillus niger* Environmental Isolates

A comparison of the biochemical profiles of *A. niger* isolates shows that the oxidation of a number of substrates is consistent (Table 2). The same positive reaction was found when utilising the following substrates: Tween 80, N-acetyl-D-glucosamine, amygdalin, L-arabinose, arbutin, dextrin, α-D-glucose, glycerol, glycogen, D-mannose, D-raffinose, D-ribose, salicin, D-sorbitol, D-xylose, quinic acid, D-saccharic acid, L-alanine, L-alanyl-glycine, L-asparagine, L-glutamic acid, and L-proline. The same negative reaction was observed when utilising the following substrates: N-acetyl-D-galactosamine, N-acetyl-β-D-mannosamine, adonitol, D-arabinose, D-arabitol, β-cyclodextrin, L-fructose, D-galactose, D-galacturonic acid, D-glucosamine, glucuronamide, α-D-lactose, lactulose, maltitol, palatinose, sedoheptulosan, D-tagatose, bromosuccinic acid, β-hydroxy-butyric acid, α-keto-glutaric acid, D-lactic acid methyl ester, L-lactic acid, sebacic acid, succinic acid, N-acetyl-L-glutamic acid, L-alaninamide and adenosine-5′-monophosphate. These biochemical tests, in which identical positive or negative results were obtained for all examined *A. niger* isolates, were not included in the phenotypic comparison of the strains.

**TABLE 2:** Comparison of the results of phenotypic (biochemical) identification profiles of *Aspergillus niger* environmental isolates obtained by the Biolog system.

Positive reactions		
(A2) Tween 80	(C4) Glycerol	(E12) D-Xylose
(A4) N-Acetyl-D-Glucosamine	(C5) Glycogen	(F12) Quinic Acid
(A7) Amygdalin	(D2) D-Mannose	(G1) D-Saccharic Acid
(A9) L-Arabinose	(D11) D-Raffinose	(G8) L-Alanine
(A11) Arbutin	(E1) D-Ribose	(G9) L-Alanyl-Glycine
(B3) Dextrin	(E2) Salicin	(G10) L-Asparagine
(B12) α-D-Glucose	(E4) D-Sorbitol	(G12) L-Glutamic Acid
		(H4) L-Proline

**Negative reactions**		

(A3) N-Acetyl-D-Galactosamine	(B11) D-Glucosamine	(F4) β-Hydroxy-butyric Acid
(A5) N-Acetyl-β-D-Mannosamine	(C2) Glucuronamide	(F7) α-Keto-glutaric Acid
(A6) Adonitol	(C8) α-D-Lactose	(F8) D-Lactic Acid Methyl Ester
(A8) D-Arabinose	(C9) Lactulose	(F9) L-Lactic Acid
(A10) D-Arabitol	(C10) Maltitol	(G2) Sebacic Acid
(B2) β-Cyclodextrin	(D9) Palatinose	(G4) Succinic Acid
(B6) L-Fructose	(E3) Sedoheptulosan	(G6) N-Acetyl-L-Glutamic Acid
(B7) D-Galactose	(E8) D-Tagatose	(G7) L-Alaninamide
(B8) D-Galacturonic Acid	(F2) Bromosuccinic Acid	(H12) Adenosine-5′-Monophosphate

**Different reactions**		

(A12) D-Cellobiose	(D5) α-Methyl-D Galactoside	(F6) p-Hydroxyphenylacetic Acid
(B1) α-Cyclodextrin	(D6) β-Methyl-D Galactoside	(F10) D-Malic Acid
(B4) i-Erythritol	(D7) α-Methyl-D Glucoside	(F11) L-Malic Acid
(B5) D-Fructose	(D8) β-Methyl-D-Glucoside	(G3) Succinamic Acid
(B9) Gentiobiose	(D10) D-Psicose	(G5) Succinic Acid Mono-Methyl Ester
(B10) D-Gluconic Acid	(D12) L-Rhamnose	(G11) L-Aspartic Acid
(C1) α-D-Glucose -1-Phosphate	(E5) L-Sorbose	(H1) Glycyl-L-Glutamic Acid
(C3) D-Glucuronic Acid	(E6) Stachyose	(H2) L-Ornithine
(C6) m-Inositol	(E7) Sucrose	(H3) L-Phenylalanine
(C7) 2-Keto-D-Gluconic Acid	(E9) D-Trehalose	(H5) L-Pyroglutamic Acid
(C11) Maltose	(E10) Turanose	(H6) L-Serine
(C12) Maltotriose	(E11) Xylitol	(H7) L-Threonine
(D1) D-Mannitol	(F1) γ-Amino-butyric Acid	(H8) 2-Aminoethanol
(D3) D-Melezitose	(F3) Fumaric Acid	(H9) Putrescine
(D4) D-Melibiose	(F5) γ-Hydroxy-butyric Acid	(H10) Adenosine
		(H11) Uridine

The physiological analysis of *A. niger* isolates was carried out on the basis of biochemical test results, in which different reactions were found between strains (Table 2). All biochemical test results in which *A. niger* isolates differed in the result (positive reactions, negative reactions, non-identical—ambiguous reactions) were included in this comparison.

**FIGURE 5:**
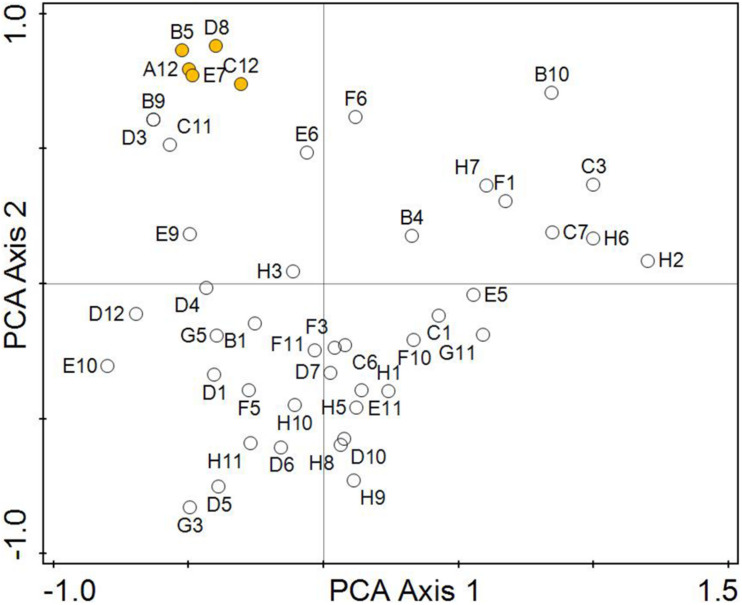
Analysis of the suitability of FF-biochemical tests from the BIOLOG system for testing the phenotypic properties of *Aspergillus niger* isolates in relation to pH of environment. The designation of the biochemical tests corresponds to Table 2. The orange centroids correspond to sugar utilisation tests (A12-B5-C12-D8-E7), these tests are best matched the pH of the original environment.

**FIGURE 6:**
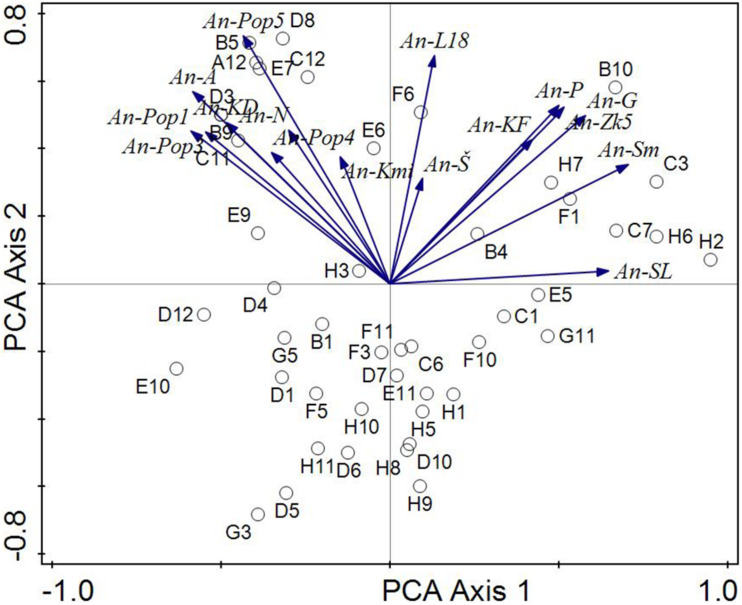
Analysis of biochemical similarity of *Aspergillus niger* environmental strains in relation to pH values of the environment. The phenotype similarity is expressed by the angle between individual strains (arrows). The correlation between the strains is stronger the sharper the angle between them. Environmental variables are shown like centroides. Direction of the arrow corresponds to the steepest increase in the covariance of *Aspergillus niger* strains and environmental variables.

In order to analyse the phenotypic similarity of strains based on biochemical parameters obtained by the Biolog system, a multicriteria analysis was used in the Canoco 5 program (Braak and Smilauer, 2012). First, the length of the gradient for the response data was calculated. Based on the calculated gradient of DS = 1.6, where a linear response to the environmental gradient is assumed, Principal Component Analysis (PCA) was selected for component analysis. Input data to the PCA formed two matrices (tables). The first matrix consisted of presence of An strains (columns) in the used biochemical tests of the Biolog system (rows). Presence was coded according to the result of Biolog-biochemical tests by system 2: positive reaction, 1: variable reaction, 0: negative reaction. The second matrix consisted of tested An strains (columns) and the corresponding pH according to used biochemical tests of the Biolog system (rows). The first two ordination axes shown in the ordination diagrams of the PCA analysis (Figures 5, 6) explain about 48% of the data variability (Table 1).

The greatest phenotypic similarity was confirmed between strains isolated from a strong alkaline environment (8.5–9.0), followed by strains isolated from a slightly alkaline, medium alkaline and strong alkaline environment (7.4–7.8, 7.9–8.8, 8.5–9.0) and strains isolated from a strong acidic (5.1–5.5) and a slightly alkaline environment (7.4–7.8). The ordinance diagram (Figure 5) did not confirm the phenotypic similarity between *A*. *niger* strains isolated from ultra-acidic (<3.5), extreme acidic (3.5–4.4), and very strong acidic (4.5–5.0) environments.

Figure 5 visualises the suitability of the FF-biochemical tests from the Biolog system for testing the phenotypic properties of *Aspergillus niger* isolates in relation to pH. The resulting data show that the most useful were the tests of the utilisation of sugars Results from the biological profile and PCA analysis showed that the sugar utilisation tests (D-Cellobiose—D-Fructose—Maltotriose—β-Methyl-D-Glucoside—Sucrose), are best matched the pH and parameters of the original environment. Tests on sugar derivatives (m-Inositol—α-Methyl-D-Glucoside—Xylitol—D-Psicose), the amino acid test of 2-Aminoethanol and tests of the utilisation of organic acids (Fumaric Acid—L-Malic Acid—Glycyl-L-Glutamic Acid—L-Pyroglutamic Acid) were usable too, but require further analysis.

The ordination diagram (Figure 6) is a summary biplot with all strains (included strains with a weak bond to environmental variables. Figure shows the *A. niger* isolates tested and their inclination to centroids representing biochemical similarity tests from the Biolog system and pH (they formed the environment variables). From these results, the An-Pop 3 and An-Pop 1 isolates (different pH of the substrate but the same isolation area of all four *A. niger* strains, namely the Poproč locality) proved to be phenotypically closest. The highest dependence was found between strains An—Pop1, An-A, and An—Pop3, this corresponds to the fact that An-Pop1 and An-Pop3 come from the same site Poproč heavily contaminated with As (and other elements). An—A was isolated from the surface of adamite [Zn_2_(AsO_4_)(OH)]—also a substrate with a high content of As. Phenotypic similarities were also found among the An-G and An-ZK isolates (both were obtained from a slightly alkaline environment). Strong similarity is further between An-P, An-KF, and An-ZK5 also from different pH of substrate and from different isolation area. The similarity between the An-A (artificial substrate) and An-KD strains is interesting too, it is probably due to the alkalinity of the substrate.

The very high similarity between the An-A and An-N isolates is of interest. The common feature of the origin of both strains is the presence of arsenic. Strain An-A was isolated from the surface of artificial adamite [Zn_2_(AsO_4_)(OH)] and strain An-N was derived from coal dust with 400 mg/kg of As from the Nováky mine. Our findings also correspond with Choe et al. (2012) which developed a fungal biosensor for arsenic in Aspergillus niger as an effective methods of detection of arsenic contamination because this strain contains seven putative arsenic metabolism and transport genes. There is also a strong similarity between the An-N and An-Pop5 strains also in connection with As contamination. The very slight similarity of the An-Pop4 strain with the other strains taken from the Poproč locality is interesting. In contrast, isolates An-SL and An-L18 appeared to be phenotypically most distant, with a slight similarity to the An-S strain. The results correspond to the extreme conditions and chemical characteristics of the sites from which the *A. niger* environmental isolates were obtained (Table 1).

The results also showed a correlation of pH values of soils and solid substrates, which correlate mainly with the biological profile of the tested *A*. *niger* environmental isolates. From the genotypic point of view, the An-SL and An-L18 isolates showed the low similarity, which was also confirmed by their lowest phenotypic similarity, including the values of multicriterial analysis. The An-SL isolate was obtained from Technosol at the Slovinky locality, which features fine-grained industrial material originated from iron ore mining enriched with many toxic elements with exceeded values, such as, Cu (8,186 mg/kg), Zn (25,108 mg/kg), Pb (2,964 mg/kg), Mn (2,647 mg/kg), and Cd (8.76 mg/kg) (Šimonovičová et al., 2017). The An-L18 strain was isolated from sludge from oil refining and other chemical processing from Ostrava. From this point of view, the slight similarity of the An-L18 strain with the An-S strain is also very interesting, because An-S was isolated from ultra-acidic Dystric Cambisol (contaminated and eroded) without vegetation (Šimonovičová et al., 2013, 2019).

The results presented in Figure 6 replicate the pH limits of the extreme environment of the occurrence of tested *A. niger* isolates in the areas (range) of ultra-acidic (An-S), neutral (An-L18) and (up to) strong alkaline (An-SL) environments.

The An-L18 isolate (Table 1) differs significantly from all other *A. niger* isolates. Only 13 species of microscopic filamentous fungi and yeast have been obtained from the sludge of the Ostrava Lagoons (Vašinková et al., 2017), and it is clear that the high content of organic pollutants in the environment inhibits the vital functions (as growth, reproduction and metabolic activity) of indigenous microorganisms and leads to a significant reduction of their biodiversity compared to the microbiota of uncontaminated sites (Doolotkeldieva et al., 2018).

The An-L18 isolate showed significant phenotypic differences in the tested utilisation reactions, which involve different enzymatic activity, gene expression and thus the formation of secondary metabolites. These likely changes can be due to the high level of tolerance to polyaromatic hydrocarbons (PAH), which has been previously confirmed to be associated with the change in morphology, production of pigments and sporulation activity of microscopic filamentous fungi in contaminated substrates containing large amounts of heavy hydrocarbon fractions (Zafra et al., 2015).

Selvig and Alspaugh (2011) studied the signalling pathways of microscopic filamentous fungi responsive to the pH of the environment and found some degree of signal specificity within the studied species. In general, if organisms grow over a wide pH range, they also adapt their gene expression to their environment. Microscopic filamentous fungi are known to be versatile, adaptable organisms, where their ability to grow over a wide pH range is conditioned by their genetic regulatory system, which adapts gene expression to the pH of the environment (Šimonovičová et al., 2013). The basis for this adaptation is primarily the metabolic link between pH and cellular nutritional enzymes, which are primarily secreted at pH values at which they can function effectively. Essential genes whose expression is influenced by the pH of the environment include genes encoding intracellular enzymes, permease, and enzymes directing the synthesis of secreted metabolites (Peñalva and ArstJr., 2002). The effect of low pH on the *Aspergillus* spp. genome was also studied by Freitas et al. (2011), who investigated acid adaptation in conjunction with the acid shock response. Analysis of the gene expression for heat shock proteins showed that these genes are expressed in an immediate response to an acidic environment. Genes whose expression has been implicated in the adaptation to acidic environments have also been implicated in the intracellular pathways of metal metabolism (Eisendle et al., 2004).

The pH regulating system ensures that secreted enzymes (e.g., alkaline and acid phosphatases, xylanases) are produced under pH conditions in which they are fully functional physiologically. Research in this field suggests that while under acidic growth conditions metabolic pathway enzymes that break down sugars (e.g., xylanase, arabinofuranosidase) are preferentially secreted, under alkaline growth conditions proteases are preferentially expressed (Katz et al., 1994; van Kuyk et al., 2000). Thus, microscopic filamentous fungi are capable of differentially producing mixtures of extracellular enzymes in response to the pH of the environment. This explains the differences in genotypic and phenotypic similarity of the tested *A. niger* strains compared to the pH of the original environment.

pH is generally acknowledged to be the principal factor governing concentrations of soluble and for soil organisms available metals (Rieuwerts et al., 1998). Metal solubility increase at lower pH and decrease at higher pH values. According to Rutkowska et al. (2015) the total concentration and activity of Zn in the soil solution increased in the soil and/by rising soil acidity. Bang and Hesterberg (2004) confirmed higher mobilisation of metals such as Zn, Cd and Pb in extreme acidic soils (pH 3.6). At acidic pH, more protons (H^+^) are available to saturate metal-binding sites; therefore metals are less likely to form insoluble precipitates with phosphates when the pH of the system is lowered because much of the phosphate has been protonated (Hughes and Poole, 1991; Olaniran et al., 2013). Significant correlations between soil pH and concentrations of heavy metals (Cu, Zn, Pb, Cd) was found also by Khaledian et al. (2017). The bioavailability of metals such as Cu, Zn, Ni, Cd and Pb is markedly reduced in soils with alkaline pH (Han, 2007).

The physiological profile of microorganisms obtained by the Biolog system provides valuable information on the physiological properties of the strains in addition to species-level identification and thus virtually complements their genotypic identification profile obtained by molecular techniques (e.g., 16S ribosomal RNA gene sequencing based on polymerase chain reaction). The use of GENIII, FF, and YT-microassay Biolog allows extensive metabolic profiling and authentication of various microorganisms—fungi, yeasts, and bacteria—including those that are anaerobic or microaerophilic. It is a suitable test system for determining the level of a cell’s metabolic and chemical sensitivity and provides information with a view to new antimicrobial compounds and other types of drugs useful in cell therapies and epidemiological practice in the search for potential disease reservoirs.

The Biolog system is also proving to be a very useful tool for studying the microbiome. In addition to its basic use in identifying indigenous microorganisms, it can also be used as an indicator of the succession of microbial communities, as it appears to be an appropriate testing system for understanding the vast diversity of microorganisms not only within the community structure but also for their function when interacting with the environment. According to Stefanowicz (2006), the Biolog technique is suitable for assessing environmental risks and environmental quality and, in conjunction with Ecoplate tests, it provides a sensitive and reliable index of environmental change (Gryta et al., 2014).

### Mycotoxin Profile

Ochratoxin (OTA) production was proven by the retention factors according to Filtenborg et al. (1989). The strain An-Sm yielded the spot with R_*F*_ related to RF of griseofulvin (the standard) = 0.31 in its biomass, and the strain An- L18 brought the spot with R_*F*_ = related to the RF of griseofulvin = 1.25, so, the mycotoxin OTA was produced into the strain cultivation medium. Ochratoxin A (C_2__0_H_1__8_C_*l*_NO_6_) is considered to be one of the most threatening mycotoxins to human and animal health, contaminating especially foods/feeds (Perrone et al., 2017).

Fumonisin B1 (FB1) was not produced by any of the 16 *Aspergillus niger* strains tested. Fumonisins are synthesised with great predominance by *Fusarium* spp. (Beccari et al., 2020; Stępieñ, 2020). *A. niger* is able to form mainly fumonisin B2 (FB2), especially on the cultivation media RC (reinforced clostridial agar), CYA (Czapek yeast extract agar) and YES (yeast extract sucrose medium), while usually not on the vast majority of plant-based media, such as MEA (malt extract agar base w/mycological peptone), OA (oat agar), or PDA (potato-dextrose agar). Production of FB1 was not observed on any of these named cultivation media used in the study by Frisvad et al. (2007). Our own findings tend towards the same direction—our strains were grown on MEA.

Susca et al. (2016) stressed the essential role of the presence of the genes necessary to synthesise mycotoxins in particular strains/isolates of (potentially) toxic fungal species. Their results indicated that strains of *A. niger* not producing fumonisins possess a deficient cluster of fumonisin-biosynthesis genes. The same is true in the case of non-ochratoxic isolates of *A. niger*, which do not possess a functional OTA biosynthetic genes cluster (Susca et al., 2016). Upon comparing several studies, OTA production occurred in 4–31% of *A. niger* strains, fumonisins in 63–76% of them. Differences in production of these toxins may be affected by ecologic factors, like the plant species that the fungi parasite on, the locality, climate or agricultural practices in the particular region (Storari et al., 2012; Susca et al., 2014, 2016; Massi et al., 2016).

The role of mycotoxins in soil has not been reliably clarified yet, as there is an enormously rich mixture of all kinds of metabolites present in the natural reservoir of the microorganisms. Nevertheless, it is assumed that they are important for strengthening the defence reactions of their producers towards other soil (micro)organisms, such as amoebas, nematodes, etc., which are often strong antagonists of fungi (Fox and Howlett, 2008; Karlovsky, 2008; Rohlfs and Churchill, 2011). Mycotoxins perhaps act as soil chemical signals among microorganisms (Rohlfs and Kürschner, 2010).

## Conclusion

Using the Biolog test system to investigate and understand the metabolic diversity of microorganisms directly related to their genetic profile is an interesting approach to determining their biological profile as a specific identification characteristic. It has been shown that the biological profile of a strain obtained by FF-MicroPlate can be used to assess the level of metabolic properties even between strains of a single microbial species, and their unique fingerprints can be used to detect and evaluate changes in strain adaptability in relation to a particular environmental variable.

This study compares the biological profiles of 16 *Aspergillus niger* environmental isolates. Strains obtained from different pH sites affected by anthropogenic sources *in situ* showed significantly higher metabolic similarity. The above-mentioned biological analysis was also found to be useful in distinguishing metabolic diversity between *Aspergillus niger* environmental isolates, both in relation to environmental factors that most affect phenotypic similarities and in identifying which substrates were most used by the strains. It appears that the Biolog testing system may be a suitable ecological indicator of the interactions of physicochemical changes in an environment in relation to the adaptation of microorganisms to its pH. Detailed knowledge on microscopic fungal metabolism can also provide insight into the complex interactions between microscopic fungi and possible physiological pathways that lead to their mechanisms of adaptation to environmental conditions. Environmental microscopic fungi originated at sites affected by toxic compounds may acquire genetic alterations, which may result in changes of their physiology, including the production of different (secondary) metabolites, such as mycotoxins.

## Data Availability Statement

The datasets presented in this study can be found in online repositories. The names of the repository/repositories and accession number(s) can be found below: GenBank, MW739953-MW739968.

## Author Contributions

AŠ and HV isolated all the wild fungal strains. EP and SN did the mycotoxin analyses. LK and DP did the DNA sequencing of the strains. HD did the RAMP analyses. SN, HV, HŠ, and KK did the analyses of the fungal metabolites. HŠ, HV, and BS prepared the multicriterial analysis. SN prepared the microscopic analyses, and photo and graphic documentation. AŠ, HV, EP, and DP prepared the manuscript. All authors have read and agreed to the published version of the manuscript.

## Conflict of Interest

The authors declare that the research was conducted in the absence of any commercial or financial relationships that could be construed as a potential conflict of interest.
