# Supplementary material for: Aspergillus niger Environmental Isolates and Their Specific Diversity Through Metabolite Profiling
Source: Front Microbiol. 2021 Jun 23;12:658010. doi: 10.3389/fmicb.2021.658010 (PMC8261049; doi:10.3389/fmicb.2021.658010)
Supplement: Supplementary file 1 [file Table_1.DOCX]

**Supplementary Material**

***Aspergillus niger* environmental isolates and their specific diversity through metabolite profiling**

**Alexandra Šimonovičová^1^, Hana Vojtková^2^, Sanja Nosalj^1^, Elena Piecková^3^, Hana Švehláková^2^, Lucia Kraková^4^, Hana Drahovská^5^, Barbara Stalmachová^2^, Kateřina Kučová^2^, Domenico Pangallo^4*^**

*^1^Department of Soil Science, Faculty of Natural Sciences, Comenius University in Bratislava, Ilkovičova 6, 842 15, Bratislava, Slovak Republic*

*^2^Department of Environmental Engineering, Faculty of Mining and Geology, VŠB – Technical University of Ostrava, 17. listopadu 2172/15, 708 00 Ostrava – Poruba, Czech Republic*

*^3^Slovak Medical University in Bratislava, Limbová 12, 833 03 Bratislava, Slovak Republic,*

*^4^Institute of Molecular Biology, Slovak Academy of Sciences, Dúbravská cesta 21, 845 51 Bratislava, Slovak Republic*

*^5^Department of Molecular Biology, Faculty of Natural Sciences, Comenius University, 842 15 Bratislava, Slovak Republic*

**Correspondence:* [*domenico.pangallo@savba.sk*](mailto:domenico.pangallo@savba.sk)

**Data of PCA method**

Total variation is 330.15217

**Summary table**

| Statistic | Axis 1 | Axis 2 | Axis 3 | Axis 4 |
| --- | --- | --- | --- | --- |
| Eigenvalues | 0.2515 | 0.2312 | 0.0868 | 0.0831 |
| Explained variation (cumulative) | 25.15 | 48.27 | 56.96 | 65.27 |

**Additional results**

Single value statistics

| Statistic | Value |
| --- | --- |
| Occurrences | 531 |
| Total variation | 330.152 |
| Tau | 0.669758 |
| All eigenvalues | 1 |

Per - axis statictics

| Statistic | Axis 1 | Axis 2 | Axis 3 | Axis 4 |
| --- | --- | --- | --- | --- |
| Origin scores | -0.10808 | -2.68488 | -0.30608 | -0.34913 |

**Summary table of PCA analyses from Figure 5 and Figure 6**

**Summary table N^o^1 and N^o^2:** Summary of the principal component analysis (PCA) showing the suitability of FF-biochemical tests from the Biolog system for testing the phenotypic properties of *Aspergillus niger* isolates in relation to pH (Figure 5) and relation between environmental variables (biochemical similarity, pH) and phenotypical similarity of *Aspergillus niger* strains (Figure 6).

Summary tables were generated from CANOCO 5 notebooks with PCA results and their visualization is in the ordination diagrams (Figure 5 and 6). The Figures 5 and 6 were created from both presented summary tables (1, 2), only different visualization attributes were selected. For showing the results of the analysis of the suitability of biochemical tests graph “Plots scatter plot, axes 1 and 2” was chosen (Figure 5). For showing the relationship between environmental variables (biochemical similarity, pH) and phenotypical similarity of *Aspergillus niger* strains graph “Species + plots diagram, axes 1 and 2”was chosen (Figure 6).

**Summary table N^o^1**

**Explanatory notes**

**CaseR.1-CaseR.4** - position of cases on ordination axes, derived from response variable positions.

**CaseW** - Case weights - in linear methods these are the weights set by the user.

**CaseN2** - Hill´s measure of diversity, representing number of "effective numbers of species" in compositional data.

**SqL.1 - SqL.4** - Residual squared lenght of the case after fitting first N ordination axes.

**SqL.tot** - represents squared lenght of a case, representing of null model (with no ordination axes). This statistic can be used to estimated percentage fit per axes.

**FitC4** - Estimated fit of each of values (position of ordination space) by the first four ordination axes.

**Summary table N^o^2**

**Explanatory notes**

**Resp.1-Resp.4** - positions of response variables (e.g isolates) on ordination axes: in linear methods represent regession coefficients.

**Resp.W** - weights of response variables, in linear methods these are the weights set by the user.

**RespN2** - number of species "effective occurrences" in compositional data.

**CFit1-CFit4** - represents cummulative fraction of variation in individual response variables, explained (fitted) by the firt, first two,…etc axes. With covariates in analysis, this fit is in addition to the variation explained by the covariates.

**VarR** - variance of individual response variables (after transformation and after a centering / standardization in linear methods).
